# 77 Impacts of Financial Assistance on Quality of Life Among People Living with Burn Injury

**DOI:** 10.1093/jbcr/irac012.080

**Published:** 2022-03-23

**Authors:** Flora Martz, Kara McMullen, Gretchen J Carrougher, Steven E Wolf, Jeffrey C Schneider, Barclay T Stewart, Clifford C Sheckter, Aaron E Bunnell

**Affiliations:** Oakland University William Beaumont School of Medicine, Southfield, Michigan; University of Washington, Portland, Oregon; Department of Surgery, The University of Washington, Seattle, Washington; University of Texas Medical Branch at Galveston, Galveston, Texas; Massachusetts; University of Washington, Seattle, Washington; Stanford University, Stanford, California; University of Washington, Seattle, Washington

## Abstract

**Introduction:**

Financial toxicity negatively impacts recovery after injury. Financial assistance (FA; e.g., disability income, food stamps, low-income housing voucher) may mitigate the impacts of financial toxicity. We aimed to describe FA after burn injury and its association with health-related quality of life (HRQL) and return to work.

**Methods:**

Data from adult participants participating in a multicenter longitudinal database from 2015 to 2021 were used for complete-case analysis. Participants were separated into two groups: those who received any form of financial assistance due to their burn injury, and those who did not. The cohort and FA were described. Multi-level, mixed-effects, linear regression was performed to assess the associations of FA with VR-12 Physical and Mental Health Component Summary scores (PCS, MCS) and return to work. Lastly, a propensity score analysis matched 3:1 on age, gender, pre-injury PCS and MCS, burn size, length of hospital stay, and the number of operations as a result of burn injury was used to maximally reduce potential confounding.

**Results:**

The analysis included 1,237 participants [725 who received FA, 512 who did not receive FA (NFA)]. Participants who received FA due to their burn injury were more likely to be younger (median 42 FA vs 48 NFA, p-value < 0.001), racially minoritized (19.2% FA vs 14.3% NFA, p-value < 0.001), have larger injuries (21% FA vs. 10% TBSA NFA, p-value < 0.001), longer hospital stays (median 29.5 days FA vs. 17 days NFA, p-value < 0.001), more days before returning to work (median 220 days FA vs 79 days NFA, p-value < 0.001), and have a workers compensation insurance payer (23.6% FA vs. 9.38% NFA, p-value < 0.001) compared to peers who did not receive FA. The number of participants who received new FA decreased after the 6-month time point: 11% at discharge, 33% at 6 months, and 15% at 12 months. Propensity score analysis demonstrated that receiving FA was associated with lower PCS and MCS scores at all time points and longer time to return to work (Table 1).

**Conclusions:**

Given that financial toxicity is associated with unsatisfactory recovery after injury, efforts to reduce financial stressors are needed. FA seems somewhat matched to patients with greater recovery challenges (e.g., larger injuries, more complex hospitalizations). Additionally, most patients do not receive FA for a prolonged period (e.g., >6 months). While FA is associated with lower HRQL and longer return to work, these data may represent improvement compared to what people living with burn injury might have experienced without FA and represent unmeasured confounding.

